# Adaptation to numerosity affects the pupillary light response

**DOI:** 10.1038/s41598-024-55646-w

**Published:** 2024-03-13

**Authors:** Camilla Caponi, Elisa Castaldi, David Charles Burr, Paola Binda

**Affiliations:** 1 Department of Neuroscience, Psychology, Pharmacology and Child Health, University of Florence, Florence, Italy; 2 Department of Translational Research on New Technologies in Medicine and Surgery, University of Pisa, Pisa, Italy

**Keywords:** Sensory processing, Object vision, Human behaviour

## Abstract

We recently showed that the gain of the pupillary light response depends on numerosity, with weaker responses to fewer items. Here we show that this effect holds when the stimuli are physically identical but are perceived as less numerous due to numerosity adaptation. Twenty-eight participants adapted to low (10 dots) or high (160 dots) numerosities and subsequently watched arrays of 10–40 dots, with variable or homogeneous dot size. Luminance was constant across all stimuli. Pupil size was measured with passive viewing, and the effects of adaptation were checked in a separate psychophysical session. We found that perceived numerosity was systematically lower, and pupillary light responses correspondingly smaller, following adaptation to high rather than low numerosities. This is consistent with numerosity being a primary visual feature, spontaneously encoded even when task irrelevant, and affecting automatic and unconscious behaviours like the pupillary light response.

## Introduction

All animals, from humans to invertebrates, spontaneously perceive the number of objects or individuals in a visual scene^1–3^. Electrophysiological studies in monkeys and crows have identified dedicated neural detectors that fire maximally when presented with a specific number of dots, gradually decreasing as the array numerosity deviates from the preferred^4–6^. These neurons spontaneously display numerical tuning, even if the animals had not received any specific training on numerosity tasks^5^. As for many other primary attributes of the scene, such as color, motion, orientation, and in line with the existence of number detectors, adaptation aftereffects have been reported for numerosity perception in humans: the repeated or prolonged exposure to highly numerous dot arrays, makes the subsequent arrays appear much less numerous than what they really are^7–10^.

In line with it being a primary visual attribute, numerosity appears to be encoded automatically, since very early in life. For example, studies in human babies showed that a coarse sensitivity to numerical information is present at few hours after birth^11,12^. Crucially, adults cannot ignore numerosity information during categorization, discrimination, or oddball tasks, where non-numerical characteristics of the image change concurrently^13–15^. Moreover, humans can make numerical choices by saccading towards the more numerous of two sets of dots as quickly as 190 ms^16^, indicating a spontaneous orienting response evoked by categorizing stimuli based on their numerosity, reminiscent of the spontaneous categorization of animate versus inanimate objects^17^.

Our recent study^18^ showed that numerosity information also affects the constrictions and dilations of the eye-pupil elicited by luminance increments and decrements, arguably one of the simplest visual responses, and certainly one that occurs outside our voluntary control. In that study, perceived numerosity was manipulated by the *connectedness* illusion, where numerosity is underestimated when dots are connected by lines^19–21^. In the pupillometry experiment, participants passively observed arrays of variable numerosities, with or without dots being connected, without performing any task (which may have cued participants to numerosity). Although luminance was matched across stimuli, the pupil light response scaled with the perceived numerosity of the stimuli, constricting more to white dot arrays of high numerosity, and dilating more to dark. The replication of this finding with different types of stimuli allowed us to rule out some potential confounds, such as the possibility that the pupil light response was driven by spatial frequency, convex hull, or density differences across stimuli. Here we take an even more extreme approach, by measuring the pupillary evoked response to physically identical arrays, while altering their perceived numerosity by means of adaptation.

## Methods

### Participants

The sample size was estimated from the only previous study that has measured pupillary responses to numerosity^18^. An a-priori power analyses, using G*Power 3 Software (Faul et al. 2007), with an ⍺ = 0.05 and a Power of 0.8, indicated a required sample size of 12 participants. Two experiments involved N = 12 (Exp1) and N = 13 (Exp2); three participants took part in both experiments, implying an overall sample of 22 participants (6 males, mean age: 25.7 ± 4.3 years). All participants took part in the pupillometric tests, followed by a psychophysical test (except in 4 participants who failed to complete the psychophysical test); they all had self-reported normal or corrected to normal vision. The research was approved by the local ethics committee (*Commissione per l’Etica della Ricerca*, University of Florence, n. 111 dated 7 July 2020) and was in accordance with the Declaration of Helsinki. Written informed consent was obtained from all participants prior to the study.

### Stimuli and procedures

Data were collected in a quiet dark room with participants sitting at 57 cm from the screen, with their head stabilized with a chin and front rest. The only light source was the stimulus display (LCD screen, 1920 × 1080 pixels, refresh rate 60 Hz). An EyeLink 1000 system (SR research) with infrared camera mounted below the screen was used to monitor pupil diameter of the left eye at 500 Hz. Eye position was linearized by a standard nine-point calibration routine prior to each session. Stimuli were generated and displayed through Matlab using Psychtoolbox-3 routines^22^.

Visual stimuli were arrays of bright dots centrally presented against the gray background of 129 cd/m^2^. Dots were designed to fall within a virtual circle of 11 visual degrees diameter, slightly smaller than our previous paper^18^, 14.4 deg diameter. The distribution of the items was further constrained to ensure that dots were at least 0.25 deg apart from each other and did not overlap with the fixation point.

Adaptation arrays contained either 160 (“adapt-high” condition) or 10 dots (“adapt-low” condition). The total surface area (number of bright pixels) across adapting stimuli was matched and equal to 181 deg^2^. This resulted in smaller dots for the adapt-high condition (1.2 deg diameter) compared to the adapt-low condition (4.8 deg diameter).

Test stimuli were arrays of 10, 14, 20, 28 and 40 white dots. In all cases, their total surface area was matched implying constant luminance. In two experiments, we varied individual dot sizes. In Experiment 1, dot size decreased with increasing numerosity (diameters: 3.2, 2.7, 2.3, 1.9, 1.6 deg) and it was homogeneous within each stimulus (e.g. all 10 dots of the first array had 3.2 deg diameter). The total surface area was 80.4 deg^2^ (implying an average luminance of the display screen of 136.1 cd/m^2^). In Experiment 1 smaller item size was directly associated with higher numerosities. In the attempt to disentangle numerosity from individual item size, we introduced some jitter in the item size. In Experiment 2 we created heterogeneous arrays, wherein the diameter of individual dots within each array varied by 50%. The average dot diameter scaled with numerosity and the average diameters were the same as in Experiment 1. This variability imposed small variations of total surface area across stimuli (82.6 ± 6.7 deg^2^) producing marginal variations of the average display luminance (136.3 ± 0.6 cd/m^2^). Although the total surface area values were close to our previous study (82.3 deg^2^)^18^, in that study individual item size varied less across numerosity (2.2 and 1.9 deg diameter for N18 and N24 respectively) and the luminance of white dots was higher (fixed at 256 cd/m^2^). Most importantly, our previous study presented stimuli for 6 s, much longer than in the present one (500 ms, see below).

The same stimuli were used in the pupillometry and psychophysical experiments. In the pupillometry experiment, participants passively watched the stimuli. Their only task was to maintain their gaze on the central fixation cross for the entire duration of the session. In the psychophysical experiment, participants responded to each test stimulus providing a vocal estimate of its numerosity, which the experimenter recorded using a keyboard. The pupillometry experiment was always performed before the psychophysical experiment, to avoid biasing participants’ attention towards the numerical dimension of the stimuli.

Adaptation to high and low numerosities was tested in separate sessions. Each session began with 60 s of adaptation; in addition, each trial started with a 2-s top-up adaptation. During adaptation, dots were redrawn at new random locations every 500 ms. Following the top-up, trials featured a 500 ms blank interval, followed by the presentation of the test stimulus for 500 ms. The inter-trial-interval was fixed to 2.5 s for the pupillometry experiment, and variable for the psychophysical experiment, determined by the latency of the participant’s response. For both experiments, participants performed 4 sessions (2 for each adaptation condition), resulting in 360 trials.

### Data analysis

Numerical estimates collected during the psychophysical experiment were analyzed with a Generalized Linear Mixed Model (GLMM) with numerosity estimates as dependent variable and the same two fixed factors used for the pupillometry analyses: numerosity and high/low adaptation condition. Random effects represented participants and experiment. Responses exceeding 3 standard deviations from the mean were treated as outliers and excluded from the analyses.

Pupil-size traces were preprocessed to exclude artifacts. These included unrealistic pupil sizes (smaller than 1 mm), unrealistic changes in pupil sizes (faster than 25 mm/s) and blinks. Trials in which participants made saccades with an amplitude greater than 1 deg, were eliminated (on average 5.5% of trials). Valid data points from the retained trials were down-sampled at 20 Hz.

**Figure 1:**
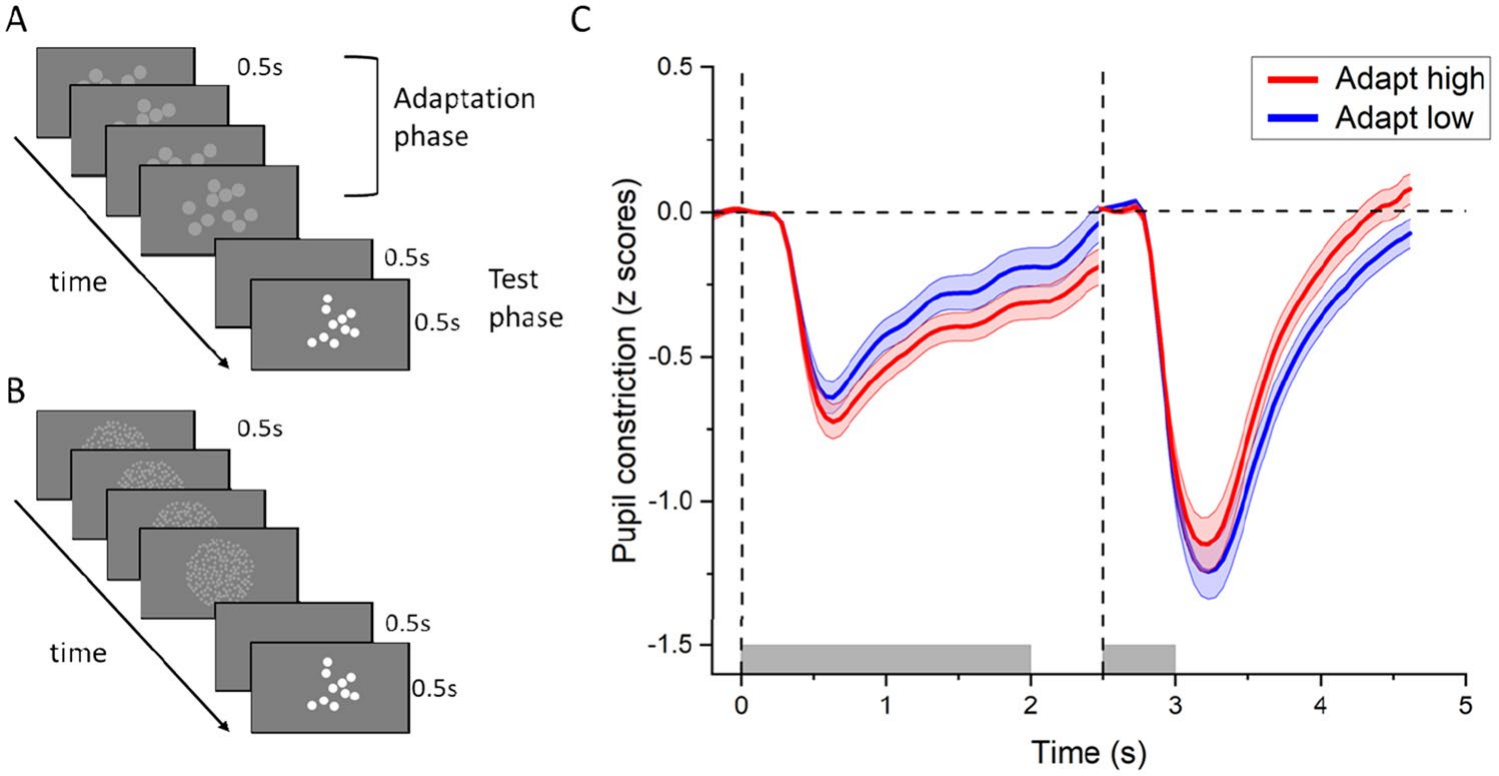
Paradigm and stimuli. (**A**, **B**) Schematic representation of a trial in the adapt low (**A**) and adapt high (**B**) conditions. After the test stimulus presentation participants simply maintained gaze on the fixation point for another 2.5 s (pupillometry experiment) or verbally estimated the numerosity of the test stimulus (psychophysical experiment) before the following trial was presented. (**C**) Time courses of pupil size in response to the adapter and test stimuli during and after adaptation to high (160 dots, red curves) and low (10 dots, blue curves) numerosities. The vertical dashed lines and the gray shaded area on the abscissa define the onset and offset of the adapting and test stimulus. Thick lines show the pupil constriction averaged across participants and across test numerosities; the shaded area represents the SEM.

To account for inter-individual variability in pupil responsivity (crucial for correlating pupillary and behavioral responses), pupil-size traces were z-scored (for each participant, the median of the pupil traces was subtracted from each data point, and then divided by the standard deviation—both median and standard deviation were computed over the entire trace). Z-scored time courses for each trial were further baseline-corrected by subtracting the median pupil diameter in the 400 ms around the onset of the adapter stimulus (leftmost part of Fig. 1C) and the same window around the onset of the test stimulus (rightmost part); this step was necessary to reduce inter-trial variability of pupil diameters at trial onset, representing the slow fluctuations of pupil diameter that are unrelated to the stimulus presentations.

Baseline-corrected traces from each trial were used to define the peak pupil constriction within a fixed a priori defined temporal window (from 0.3 to 1.3 s after the test stimulus onset, 0.3 s being the minimum latency of the pupillary response).

These values were entered a Generalized Linear Mixed Model (GLMM), with pupil size as dependent variable and numerosity (continuous variable, from 10 to 40 dots), and adaptation condition (categorical variable: high and low adaptation conditions) as fixed effects. We added a third fixed effect defined as the pre-stimulus pupil diameter (computed from − 200 to 0 ms before test appearance); this accounted for any residual pupil-size difference following the extinction of the adapter (see Fig. 1C, separation of the blue and red traces at about x = 2.5 s). Preliminary analyses revealed very similar results from Experiments 1 and 2; consequently, the main analysis pooled data from the two experiments. Random effects represented participants, experiment (1 and 2) and luminance (the marginal variations occurring in Experiment 2).

**Figure 2:**
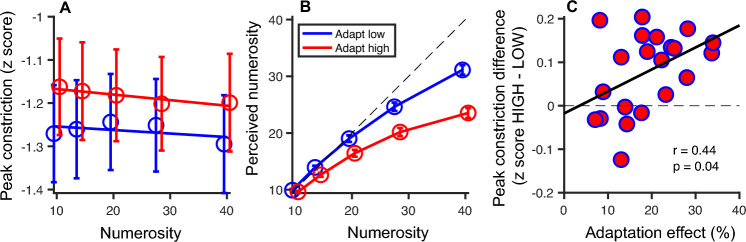
Results of the pupillometry and psychophysical experiments. (**A**) Average peak constriction in the 1 s time window around the peak for each test numerosity. Symbols represent average constriction across participants and error bars represent ± 1 SEM. (**B**) Perceived numerosity is plotted as a function of physical numerosity after adaptation to high (red curves and symbols) and low (blue curves and symbols) numerosities. Symbols represent average perceived numerosity across participants and error bars represent ± 1 SEM. (**C**) The difference between pupil traces during the two conditions (high and low) correlates significantly with the magnitude of individual perceptual adaptation.

Baseline-corrected traces were also averaged across trials, separately for the adapt-high and adapt-low conditions, and for each numerosity. Within the same a priori defined temporal window used above, we computed the peak constriction, which we used in Fig. 2A. We also averaged traces across numerosities and correlated the peak constriction difference between high- and low- adaptation conditions with the difference between numerical estimates of the highest test numerosity (40 dots, where the psychophysical effect was stronger).

## Results

Participants were adapted to low and high numerosities (Fig. 1A,B) to alter numerosity perception of subsequently presented stimuli.

Pupil size was continuously measured while participants viewed arrays of 10 or 160 bright dots of matched luminance and contrast (same number of bright pixels) followed by arrays of various numerosities, again comprising the same number of pixels (scaling dot size inversely with numerosity). Participants passively viewed the stimuli without any task. Figure 1C shows the pupil light responses to adapter and test stimuli, each baseline-corrected to the median pupil size recorded 400 ms around the presentation of the relevant stimulus.

Although luminance was identical for all numerosities, the high-numerosity adapter elicited stronger pupillary light responses than the low-numerosity adapter, replicating our previous results^18^. And although the test stimuli were physically identical across conditions, they evoked weaker pupillary constrictions when presented after a high-numerosity adapter, consistent with their numerosity being underestimated.

We next focused on the pupillary responses to the test stimuli and quantified the effects of adaptation condition and numerosity with a Generalized Linear Mixed Model. As the 500 ms interval between the adaptor and test stimulus was not sufficient to allow pupil size to return completely to baseline, we used the pre-test pupil size to define an additional fixed factor. Both the numerosity and adaptation effects were significant, suggesting that these factors modulated the pupillary response to the test stimulus over and above the pre-test pupil size (adaptation: F(1,7380) = 16.6, *p* < 0.001; numerosity: F(1,7380) = 4.7, *p* = 0.030; pre-test pupil size: F(1,7380) = 584, *p* < 0.001). Specifically, the pupil constricted more as the numerosity of the test stimuli increased and the pupillary constriction in response to physically identical test stimuli was stronger after adaptation to low compared to high numerosity. Neither the interaction between numerosity and adaptation condition, nor that between adaptation and baseline were significant.

After the pupillometry experiment, participants performed a psychophysical experiment with the same stimuli and reported perceived numerosities in the two adaptation conditions (Fig. 2B). In line with previous studies^7–9^, we found that the perceived numerosity of the test stimuli depended on the numerosity of the adapter, with the high-numerosity adapter eliciting the strongest underestimation of the test numerosities. A Generalized Linear Mixed Model with numerosity and adaptation condition as factors confirmed the main effect of adaptation (F(1,6725) = 111, *p* < 0.001), indicating that test numerosities were significantly more underestimated after adapting to high than to low numerosity. There was also an interaction between numerosity and adaptation condition (F(4,6725) = 948, *p* < 0.001), indicating that the behavioral underestimation effect following adaptation to high versus low numerosities was strongest for the higher numerosities and decreased for lower.

Finally, Fig. 2C shows that the effects of adaptation on pupillary and behavioral responses were correlated (Pearson’s r = 0.44, *p* = 0.04), indicating that participants who showed a stronger perceptual adaptation effect also showed a stronger modulation of the pupillary light response.

## Discussion

We investigated whether the pupillary light response is modulated by perceived numerosity after adaptation. Participants passively viewed bright arrays of constant luminance and variable numerosity, following adaptation to arrays of low or high numerosity. Although participants were not asked to judge numerosity, their pupils constricted less in response to the arrays that appeared to be less numerous; importantly, this was seen even for arrays that were physically identical but were perceived as less numerous, after prior exposure of a high-numerosity adapter. These results suggest that the pupillary light response reflects perceived numerosity and its changes with adaptation.

When considering the pupillary responses to the adapter, we found a very obvious pupil modulation with numerosity (despite luminance being exactly matched across stimuli), as in our previous study. When varying the physical numerosity of an array, other non-numerical quantities necessarily change as well, and it is not possible to simultaneously control for all of them at the single trial level. In our previous studies, these were controlled for at the level of trial-averages. This was not possible in this study for the adapter-stimuli, but for the test stimuli we achieved an even more stringent level of control. We varied perceived numerosity without altering any physical property of the stimuli—just by presenting them after a low- or high-numerosity adapter. We still found a very obvious modulation of pupil size, which scaled with perceived numerosity in a way that correlated with (successively collected) psychophysical judgments. This provides strong evidence that perceived numerosity indeed affects pupillary light responses.

One limitation of our stimulus set-up concerns timing. The test stimuli appeared a full 500 ms after disappearance of the top-up adapter. This time was chosen to be short enough to allow the adapter to influence processing of the test, while being long enough to allow for a relaxation of the pupil diameter after extinguishing the adapter. Inspection of Fig. 1C shows that the latter was incompletely achieved, implying that residual pupil-size differences could in principle have inflated the difference between responses to the test-stimuli—a possibility we excluded by including pre-test pupil-size in our statistical model, which reported significant (numerosity and) adaptation effects over and above the pre-test pupil size difference between adaptation conditions.

It is striking that the pupillary light response reflects the numerosity of the arrays, even if stimuli were presented for only 500 ms, and despite some variability related to individual item size differences between arrays. Several studies have reported that numerical estimates can be biased by individual item size, although the direction and the strength of the interference varies widely across study and across participants^13,23–28^. The current study was not designed to quantify this interference, so it is possible that numerical estimates were slightly biased due to the incongruent changes in item size that might have decreased the expected pupillary modulation. However, our results do exclude that pupillary constrictions reflect only item size, as in this case we should have observed stronger constriction for the less numerous arrays, which contained larger items. Future studies should test whether the pupil light response can be triggered to reflect item size rather than numerosity if participants’ attention is cued towards that feature. Also, it would be interesting for future research to quantify the saliency of the numerosity change compared to the other visual features, using a saliency model such as Oster et al.^29^, to attempt to investigate the pupillary response to luminance, color and set size using this approach.

Unlike the behavioral effect, where the magnitude of underestimation varied directly with numerosity, adaptation affected pupillary responses similarly at all numerosities. This partial discordance is probably explained by the lower fidelity and/or reliability with which pupillometry can be expected to index perceive numerosity, compared with direct perceptual reports.

Taken together, these results suggest that the gain of the pupillary light response is modified by adaptation and reflects, within the limits imposed by the resolution of this technique, perceived numerosity.

An open question is where the perceptual effects elicited by numerosity adaptation take place and how do they modulate the pupillary light response. The neural changes induced by numerosity adaptation have been described by neuroimaging studies in humans. Several fMRI studies recorded distance-dependent signal release from numerosity habituation bilaterally along the intraparietal sulcus^30^, suggesting the existence of mechanisms tuned to perceived numerosity. At the same parietal level, another fMRI study using MVPA succeeded in decoding numerosities when the classifier was trained and tested with activity patterns recorded before adaptation, but classification accuracy dropped when tested with activity patterns elicited by the very same numerosities after adaptation, suggesting that the patterns of activity were altered by adaptation^31^. Finally, using pRF mapping a recent study found that numerosity adaptation changed the preferred numerosity within several numerosity maps, located in frontal, parietal, occipital and temporal cortices^32^. Interestingly, numerosity maps selective to haptic numerosities have been identified also in the putamen^33^, suggesting that numerosity might be encoded already in subcortical areas. These studies indicate that a large network of cortical and subcortical areas is involved in numerosity perception. An open question is whether any of these would be able to affect the control of pupillary light responses.

Although primarily driven by the amount of light hitting the retina, as well as other low level factors (such as focal distance), a large body of literature has shown that other intermediate (e.g. alerting and orienting) and high level factors modulate the pupillary response to light, even if image luminance is kept constant^34–39^. Among the high-level factors, pupillometry has been recently exploited as a method to track attention, visual illusions, imagery, and spontaneous perceptual strategies. For example, the pupils constrict or dilate more depending on whether participants attend to a bright or dark stimuli, irrespective of their spatial overlap, suggesting that it can track both feature and spatial attention^40–43^. Pupil constriction can be evoked by the implied brightness of pictures, as in images of the sun^44^, by brightness^45^ and size illusions^46^. Compelling evidence supporting the idea that pupil size is not only influenced by low level factors comes from studies that manipulate perceptual content while maintaining a constant physical stimulation. In illusory 3D stimuli, the pupil modulation tracks the polarity of the surface perceived to be in front, and the magnitude of this modulation varied with the local–global perceptual style, as measured with the by the Autism-Spectrum Quotient (AQ) questionnaire in neurotypical adults^47,48^. Other studies using binocular rivalry and interocular grouping rivalry evoking alternations between dark or bright percepts found that pupil size modulations tracked alternations between dominance phases, even though the physical stimulation did not change^49,50^.

In conclusion the current results provide clear evidence that the perceived numerical content of an image can be read out from the pupil diameter, and that the pupil light response can reflect visual aftereffects. The neural pathways mediating such modulation remain unknown; the passive viewing paradigm introduced here, dissociating perceived numerosity from the physical number of items in a display, appears to be ideally suited for further neuroimaging and neurophysiological studies aimed at tackling this question.

## Data Availability

. The results from this study may be dowloaded at: 10.5281/zenodo.10728134.
